# Ginsenoside protopanaxadiol protects adult retinal pigment epithelial-19 cells from chloroquine by modulating autophagy and apoptosis

**DOI:** 10.1371/journal.pone.0274763

**Published:** 2022-12-01

**Authors:** Haesung Lee, Anh Thu Nguyen Hoang, Sook-Jeong Lee

**Affiliations:** Department of Bioactive Material Sciences and Research Center of Bioactive Materials, Jeonbuk National University, Jeonju, Jeollabuk-do, Republic of Korea; Faculty of Medicine, University of Belgrade, SERBIA

## Abstract

Chloroquine often causes serious eye and vision problems, which are mainly mediated by lysosomotropic alteration. In this study, we investigated whether the ginsenoside protopanaxadiol relieves chloroquine-induced retinopathy by restoring lysosomotropic abnormalities in human adult retinal pigment epithelial-19 cells. Cytotoxicity was assessed using a 3-(4,5-dimethylthiazol-2-yl)-2,5-diphenyltetrazolium bromide assay. Morphological alterations in autophagosomes of adult retinal pigment epithelial-19 cells was detected using confocal microscopy. Apoptosis was examined using flow cytometry, whereas cellular reactive oxygen species levels were determined by measuring the fluorescence intensity of 5-(and-6)-carboxy-2’-7’-dichlorohydrofluorescein diacetate. Lysosomal function was assessed by measuring lysosomal pH and enzyme activity. Immunoprecipitation and western blotting analyses were performed. Adult retinal pigment epithelial-19 cells accumulated autophagosomes with fusion defects in lysosomes and reactive oxygen species formation following exposure to chloroquine. This effect trapped Beclin-1 and B-cell lymphoma 2 interfering with autophagy initiation and autophagosome development. Protopanaxadiol alleviated chloroquine-induced toxicity by modulating the interaction between Beclin-1 and Bcl-2, which was mediated by the AMP-activated protein kinase-mammalian target of rapamycin signal axis. Furthermore, autophagy and apoptosis were simultaneously controlled by protopanaxadiol via upregulation of autophagy flux and decreased reactive oxygen species formation and apoptotic protein expression. These findings suggest that protopanaxadiol is a promising treatment strategy for chloroquine-mediated retinopathy.

## Introduction

Chloroquine (CQ) is an anti-malarial and anti-inflammatory agent [1–3] and the key mechanism underlying its therapeutic efficacy is its lysosomotropic characteristics [4]. Because CQ is a small, amphiphilic, and weakly basic, it is easily transported into acidic cytosolic organelles, including endosomes and lysosomes, and increases the intra-organellar pH [5, 6]. This ultimately prevents the infectious virus from releasing its genetic material into cells. CQ is a relatively safe medicine, but it has several severe side effects, such as ocular side effects involving the retina, which are the most serious because they can cause vision changes [7].

The retinal pigment epithelium (RPE) is a single layer of cells that maintains the photoreceptor outer segment (POS) of the retina. The RPE protects the POS by clearing shed debris from rods and cones [8]. Debris endocytosed by endosome is ultimately degraded in lysosomes [8]; however, defects in the degradation of POS debris can degenerate the RPE cells.

Lysosomal biogenesis plays a key role in autophagy, which is a self-digestion process that functions as a “garbage disposal”. These functions are achieved by the selective degradation of cellular components, including long-lived proteins, protein aggregates, damaged cytoplasmic organelles, and intracellular pathogens, resulting in the recycling of nutrients and the generation of energy [9]. In addition to autophagy, cells can be degraded by apoptosis, a canonical programmed cell death pathway.

Autophagy and apoptosis are altered under various cellular stress conditions. Autophagy and apoptosis are interconnected through several molecular nodes of crosstalk, such as B-cell lymphoma-2 (Bcl-2), Bcl-extra large (Bcl-xL), Beclin-1, AMP-activated protein kinase (AMPK), and mitogen-activated protein kinase (MAPK), enabling the coordinated regulation of degradation by these pathways [10, 11].

Ginseng is widely used for medical purposes and contains a variety of active substances including saponins, essential oils, polyacetylene, phenols, glycosides, and peptides. The major components extracted from ginseng are ginsenosides, including Rg2, Rg3, Rg3, Rh1, and Rh2, which mediate many of their pharmacological effects [12]. 20-S-Protopanaxadiol (PPD), along with 20-S-protopanaxatriol (PPT), is a major metabolite of ginsenosides that is effective against various cancer cells, diabetes, and inflammation and controls blood pressure, cognitive function, and immune responses [12–15].

Recent studies have demonstrated that certain ginsenoside derivatives, such as 20(S)-ginsenoside Rg3 and PPD, exert anticancer effects that are mediated by the control of the essential cellular processes of autophagy [16] or apoptosis [17–19]. Therefore, we investigated the effect of PPD on CQ-induced toxicity in ARPE-19 cells, with a particular focus on the pathways regulating the balance between autophagy and apoptosis.

## Materials and methods

### Chemicals and antibodies

Asiatic acid (#A2612), bafilomycin A1 (BA1) (#B1793), compound C (#P5499), CQ (#C6628), ammonium chloride (NH_4_Cl, #A9434), PPD (#P0031), rapamycin (Rap, #553210), and staurosporine (#S5921) were acquired from Sigma-Aldrich (St. Louis, MO, USA). Antibodies against the autophagy marker light chain 3 (LC3) A/B (#12741), p62 (#8025), mechanistic target of Rap (mTOR) (#2983), phosphorylated (p)-mTOR (#5536), Bcl-xL (#2764), Bcl-2 (#15071), p38 mitogen-activated protein kinase (MAPK, #8690), p-p38 MAPK (#4511), p-c-Jun N-terminal kinase (JNK, #4668), JNK (#9252), AMP-activate protein kinase (AMPK, #5831), and p-AMPK (#2535) were obtained from Cell Signaling Technology (Danvers, MA, USA). Beclin-1 (#SAB1306537) and tubulin-α (#SAB2102603) antibodies were purchased from Sigma-Aldrich. The lysosomal membrane-associated protein (LAMP)-2 (#NBP222217) antibody was purchased from Novus Biologicals (Centennial, CO, USA). Horseradish peroxidase (HRP)-conjugated goat anti-mouse (#31430) and anti-rabbit (#31460) antibodies were purchased from Invitrogen (Carlsbad, CA, USA).

### Cell culture

The human ARPE-19 cell line was purchased from American Type Culture Collection (#CRL-2302, Manassas, VA, USA) and maintained in Dulbecco’s modified Eagle’s medium (DMEM)/F-12 medium (#LM002-04) supplemented with 10% fetal bovine serum (#S001-07) and penicillin-streptomycin-amphotericin B (100 U/ml, 100 μg/ml, and 250 ng/ml, respectively, #LS202-02) from Welgene (Daegu, South Korea) at 37°C in a humidified atmosphere containing 5% CO2. For the experiment, cells were grown in DMEM/F-12 without serum supplementation.

### 3-(4,5-dimethyl-thiazol-2-yl)-2,5-diphenyltetrazolium bromide (MTT) assay

Cell viability was assessed using the 3-(4,5-dimethyl-thiazol-2-yl)-2,5-diphenyltetrazolium bromide (MTT) assay. ARPE-19 cells (2.5×10^4^ cells/well) in 48-well plates were exposed to different doses of CQ (0, 10, 50, 75, and 100 μM), 100 μM CQ+PPD (0, 0.5, 1, 2, and 5 μM), 100 μM CQ+0.5 μM Rap, and Rap alone for 24 h. Then, 30 μL MTT stock solution (5 mg/ml in phosphate-buffered saline, PBS) was added to each well, followed by incubation at 37°C for 2 h. The medium was aspirated, 300 μL dimethyl sulfoxide (DMSO) was added, and the absorbance of the resultant solution was measured at 562 nm using a microplate reader (Epoch™ microplate spectrophotometer, BioTek Instruments, Winooski, VT, USA).

### Lysosomal pH assay

ARPE-19 cells grown on glass-bottomed dishes were treated with 100 μM CQ, 100 μM CQ+ 2 μM PPD, 2 μM PPD, 100 nM BA1, and 10 mM NH_4_Cl for 6h. To detect changes in lysosomal pH, cells were labelled with1 μM Lysosensor Green DND-189 (#L7535, Life Technologies-Thermo Fisher Scientific, Waltham, MA, USA), a lysosomal-specific pH-sensitive fluorescent dye, for 10 min in a humidified CO_2_ incubator, washed twice with PBS, and imaged using the fluorescein isothiocyanate channel. The intensity of DND-189 fluorescence is inversely proportional to the pH of the lysosome. Confocal images were obtained using a laser scanning microscope (LSM) 710 confocal live-cell imaging system (Carl Zeiss, Oberkochen, Germany). BA1 and NH_4_Cl were used as lysosomal pH changers.

### Lysosomal intracellular activity assay

Lysosomal intracellular activity kit (#ab234622, Abcam, Cambridge, UK) was used to measure lysosomal function with minor modifications. Briefly, ARPE-19 cells were pretreated with the vehicle or 1× Bafilomycin A1 (BA1) for 3 h. After pretreatment, cells were incubated with vehicle, the same concentration of BA1, 100 μM CQ, 100 μM CQ+2 μM PPD, 2 μM PPD for 6 h in serum-free DMEM/F12 media according to the protocol of the kit. During incubation, 15 μl self-quenched substrate per 1 ml medium was added to the cells of each treatment group. After the experiment was terminated, the cells were harvested and washed twice in 1 ml ice-cold 1× assay buffer containing the test compound at the same concentration listed above. The cell pellets were resuspended and analyzed.

For fluorescence-activated cell sorting (FACS) acquisition and analysis, cells were selected as the main population in the forward scatter versus side scatter plot to exclude dead cells and cellular debris. In the main cell population, the mean fluorescence intensity of the fluorescence channel 1 (FL1) was quantified and compared between cells treated without and with various drugs (CQ, CQ+PPD, PPD, and BA1) to determine the different intensity levels of fluorescence emitted by the self-quenched substrate (BD FACS Calibur, Becton Dickinson, Franklin Lakes, NJ, USA). The data were analyzed using FlowJo™ software (FlowJo, Ashland, OR, USA).

For fluorescence microscopy analysis, APRE-19 cells treated using the same protocol described above were fixed with 4% (v/v) paraformaldehyde. After washing the cells with PBS, they were mounted with VECTASHIELD^®^ plus antifade mounting medium with 4’,6-diamidino-2-phenylindole (VECTOR, #H-2000, Berlingame, CA, USA). Confocal images and the resulting regions of interest (ROI) were obtained using an LSM 710 confocal live-cell imaging system (Carl Zeiss).

### Green fluorescent protein (GFP)-LC3 overexpression and immunocytochemistry for puncta detection

ARPE-19 cells transiently transfected with green fluorescent protein (GFP)-LC3 were treated with 100 μM CQ with or without 2 μM PPD, fixed with 4% (v/v) paraformaldehyde (PFA), and subsequently permeabilized with PBS containing 0.2% (v/v) Triton X-100 and 1% bovine serum albumin (BSA) at room temperature. After washing with PBS, the cells were blocked for 1 h and incubated with LAMP-2 antibody at 4°C overnight. Then, the cells were incubated with an Alexa Fluor 555-conjugated goat anti-mouse IgG (H+L) secondary antibody (#A28180, Thermo Fisher Scientific) for 1 h at room temperature. The fluorescence intensity and protein localization were analyzed using a Zeiss LSM710 laser confocal microscope (Carl Zeiss). Quantification of the number and size of GFP-LC3-positive cells and colocalization in the acquired images was performed using ImageJ software (National Institutes for Health [NIH], Bethesda, MD, USA). Mean autophagosome counts from 100 cells per treatment were obtained from three separate experiments.

### Immunoprecipitation (IP) and western blotting (WB)

For the immunoprecipitation (IP) assay, Dynabeads protein A (#10001D, Thermo Fisher Scientific) was prepared according to the manufacturer’s instructions. Briefly, cell lysates were added to the Dynabeads and antibodies complex and incubated at 4°C with rotation overnight. Then, the beads were washed with 200 μL PBS plus Tween-20 (PBS-T), resuspended in 20 μL 3× sodium dodecyl sulfate (SDS) sample buffer by gentle pipetting, heated at 95°C for 5 min, and micro-centrifuged for 1 min at 14,000×*g*. The supernatants were stored at -70°C until western blotting analysis.

For western blot (WB) analysis, the protein concentrations of the drug-treated cell lysates were measured using the bicinchoninic acid (BCA) method (Thermo Fisher Scientific). Equal amounts of protein were used for the WB analysis and the protein expression was quantified using densitometric analysis of the intensity of each protein band using the ImageJ software. All experiments were repeated at least three times using cultures from different passages.

### Flow cytometry for apoptosis assay

Apoptosis was determined using an Annexin-V and propidium iodide kit (#V13241, Thermo Fisher Scientific). ARPE-19 cells treated with the vehicle, 100 μM CQ, 100 μM CQ+ 2 μM PPD, 2 μM PPD, and 0.1 μM staurosporine for 24 h were trypsinized, collected with the media, and then centrifuged at 500×*g* at 4°C for 5 min. After washing with binding buffer, the cells were centrifuged, resuspended, and stained with Alexa Fluor® 488 Annexin V and propidium iodide. The cells were then analyzed using flow cytometry (BD FACS Calibur). Staurosporine was used as a positive control for apoptotic cell death. In total, 10,000 events were recorded for each sample and the data were analyzed using FlowJo™ software (FlowJo, Ashland, OR, USA).

### Reactive oxygen species (ROS) assay

Intracellular reactive oxygen species (ROS) levels were measured spectrofluorometrically using 2’,7’-dichloroflurescein diacetate (DCFH-DA, #D6883, Sigma-Aldrich). Briefly, ARPE-19 cells were seeded in a clear bottom 96-well black plate and incubated with the vehicle, 100 μM CQ, 2 μM PPD, or 0.1 μM staurosporine for 24 h. Then, 10 μL DCFH-DA was added to the cells, followed by incubation for 45 min and then the fluorescence intensity was measured at excitation and emission wavelengths of 485 and 535 nm, respectively, using an Epoch™ fluorescence spectrometer. Finally, cells were examined under an inverted fluorescence microscope (EVOS M5000, #AMF5000, Thermo Fisher Scientific).

### Statistical analysis

All data are presented as the means ± standard deviation (SD). For multiple comparisons among groups, a one-way analysis of variance (ANOVA) followed by Fisher’s least significant difference (LSD) post-hoc test was used. Paired *t*-tests were used to analyze differences between the two groups. Statistical significance was set at *P* < 0.05.

## Results

### Protective effect of PPD against CQ-induced ARPE-19 cell death possibly mediated by autophagy

**Fig 1:**
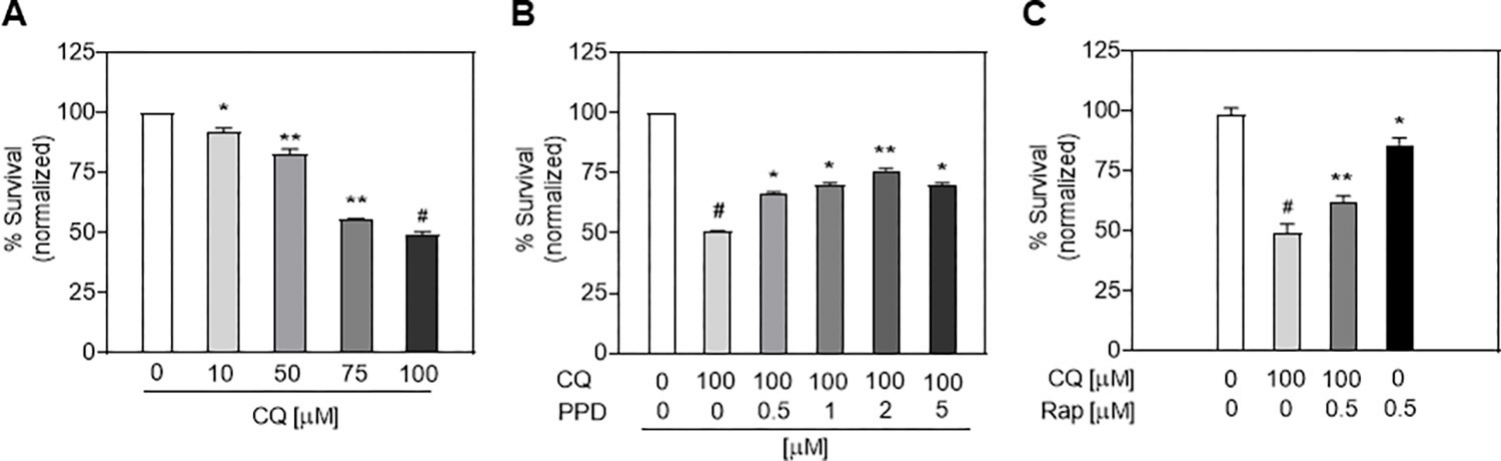
Protective effect of protopanaxadiol (PPD) on chloroquine (CQ)-induced adult retinal pigment epithelial (ARPE)-19 cell death and possible autophagy involvement. Cytotoxicity of (A) various CQ concentrations (0, 10, 50, 75, 100 μM), (B) co-treatment with 100 μM CQ and vehicle or different PPD concentrations (0.5, 1, 2, 5 μM), or (C) 0.5 μM rapamycin (Rap) for 24 h in ARPE-19 cells. Cytotoxicity was assessed using 3-(4,5-dimethyl-thiazol-2-yl)-2,5-diphenyltetrazolium bromide (MTT) assay. Mean ± standard deviation (SD), n = 3; *P<0.05, **P<0.01, and ^*#*^*P*<0.001 compared to vehicle group.

We first examined the CQ-induced cytotoxicity against our established experimental model of ARPE-19 cells. Expose of ARPE-19 cells to various concentrations of CQ (10–100 μM) resulted in a concentration-dependent decrease in the survival rate (Fig 1A), and the highest cytotoxicity (approximately 50% cell death) was observed at 100 μM. Therefore, 100 μM was used in all the subsequent experiments.

Next, we examined whether PPD, a ginsenoside, affected CQ-induced cytotoxicity in ARPE-19 cells. As shown in Fig 1B, PPD inhibited CQ-induced cell death in a dose-dependent manner, with a maximum protective effect at 2 μM. An additional cytotoxicity assay showed that CQ-induced ARPE-19 cell death was closely involved in the autophagy process, as co-treatment with CQ and Rap, a specific inhibitor of mTOR and an autophagy agonist, inhibited ARPE-19 cell death (Fig 1C).

### Recovery of lysosomal pH and function by PPD in CQ-treated ARPE-19 cells

**Fig 2:**
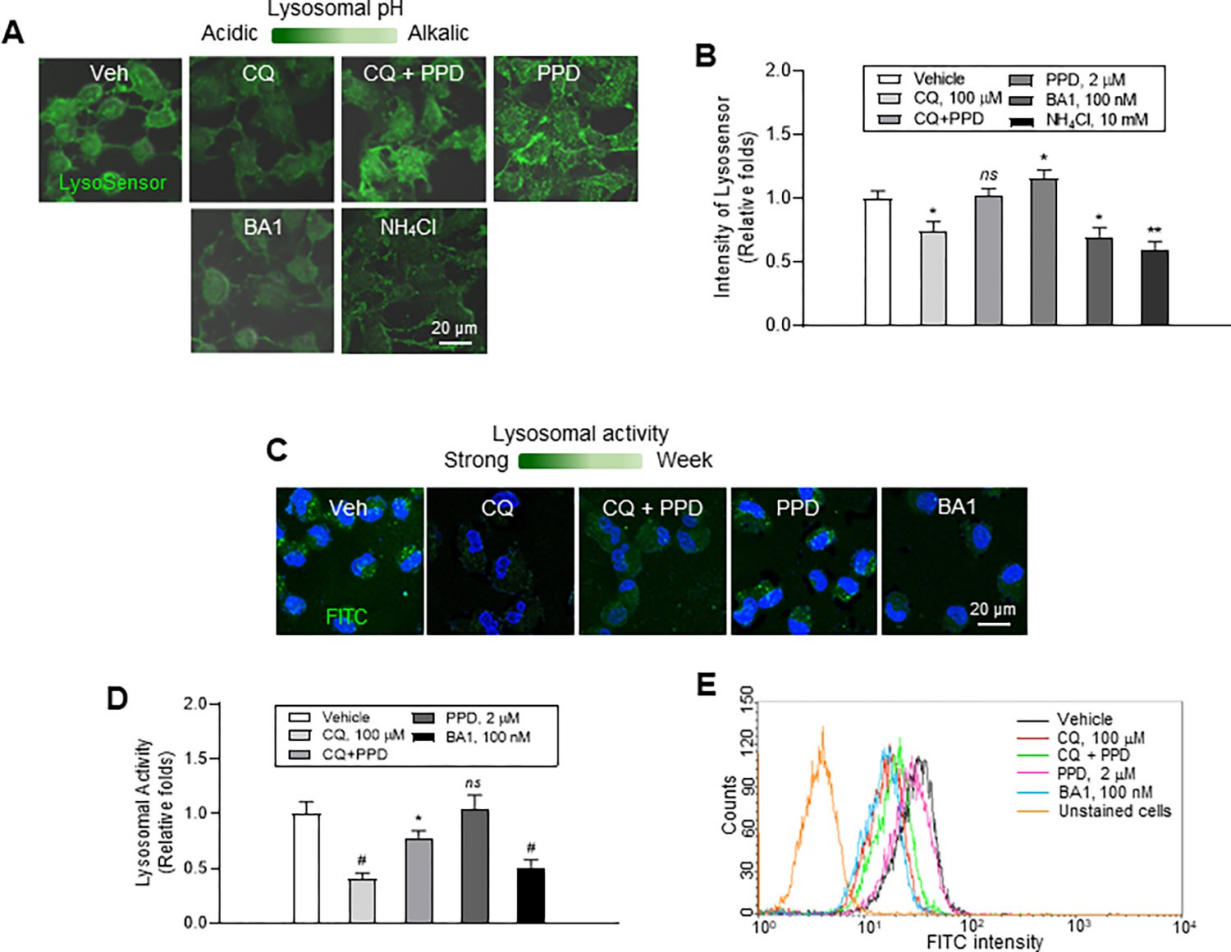
Protopanaxadiol (PPD) restored lysosomal pH and activity and chloroquine (CQ)-induced lysosomal alteration in adult retinal pigment epithelial (ARPE)-19 cells. (A) Colorimetric fluorescence images of ARPE-19 cells stained with pH-sensitive lysosomal dye, Lysosensor Green DND-189 where light green denotes relatively alkaline lysosomal pH. Cells were treated with vehicle, 100 μM CQ, CQ+PPD, and 2 μM PPD for 6 h and stained with 1 μM Lysosensor. Bafilomycin A1 (BA1, 100 nM) and ammonium chloride (NH_4_Cl, 10 mM) were used as anti-lysosomal experimental controls. (B) Bars denote relative changes in green fluorescence intensity analyzed using “Mean region of interest [ROI]” option in Zen software (n = 3–9). (C) Florescence microscopy images of ARPE-19 cells showing lysosomal intracellular activity. Cells were treated as in (A) with self-quenched substrate. Veh: untreated positive control, BA1: negative control cells. Dark green indicates stronger lysosomal intracellular activity than that shown by light green. (D) Bars show relative changes in green fluorescence intensity analyzed using “Mean ROI” option in Zen software (n = 3–9). (E) Fluorescence-activated cell sorting (FACS) acquisition and analysis of ARPE-19 cells showing lysosomal intracellular activity. Comparison of histograms of flow cytometric analysis (fluorescent channel 1/fluorescein isothiocyanate [FL1/FITC] channel), showing the inhibition of substrate de-quenching following treatment with lysosome-depolarizing agent: unstained (light brown curve), untreated positive control (black curve), CQ (red curve), CQ+PPD (green curve), PPD (crimson curve), and experimental control cells treated with 1× BA1 (blue curve). Scale bar, 20 μm. Mean ± standard deviation (SD), *ns*; not significant, n = 3; *P<0.05 and ^*#*^*P*<0.001 compared to vehicle group.

We examined whether PPD restores the CQ-induced lysosomal pH change by treating ARPE-19 cells with various combinations of drugs, followed by staining with 1 μM Lysosensor Green DND-189. As shown in Fig 2A, the positive green signal intensity of the CQ-treated cells was lower than that of vehicle treated cells, implying that CQ slightly increased the lysosomal pH. Co-treatment of cells with PPD and CQ significantly reduced the CQ-induced increase in lysosomal pH (Fig 2A and 2B), as indicated by the more intense and brighter intra-lysosomal green positive signals. PPD alone slightly reduced the lysosomal pH (Fig 2A and 2B).

Next, we examined whether CQ or PPD alter lysosomal intracellular activity, which was measured using a lysosome-specific self-quenched substrate, which has low background fluorescence, high signal-to-background ratio, and is pH insensitive. Confocal images and FACS analysis of the cells demonstrated that CQ significantly decreased the lysosomal pH, but PPD slightly increased it compared to the vehicle treatment (Fig 2C–2E). In addition, CQ+PPD treatment restored the CQ-mediated decline in lysosomal activity (Fig 2C–2E).

### PPD enhances liberation of ARPE-19 cells from CQ-mediated autophagosome accumulation and autophagy congestion

**Fig 3:**
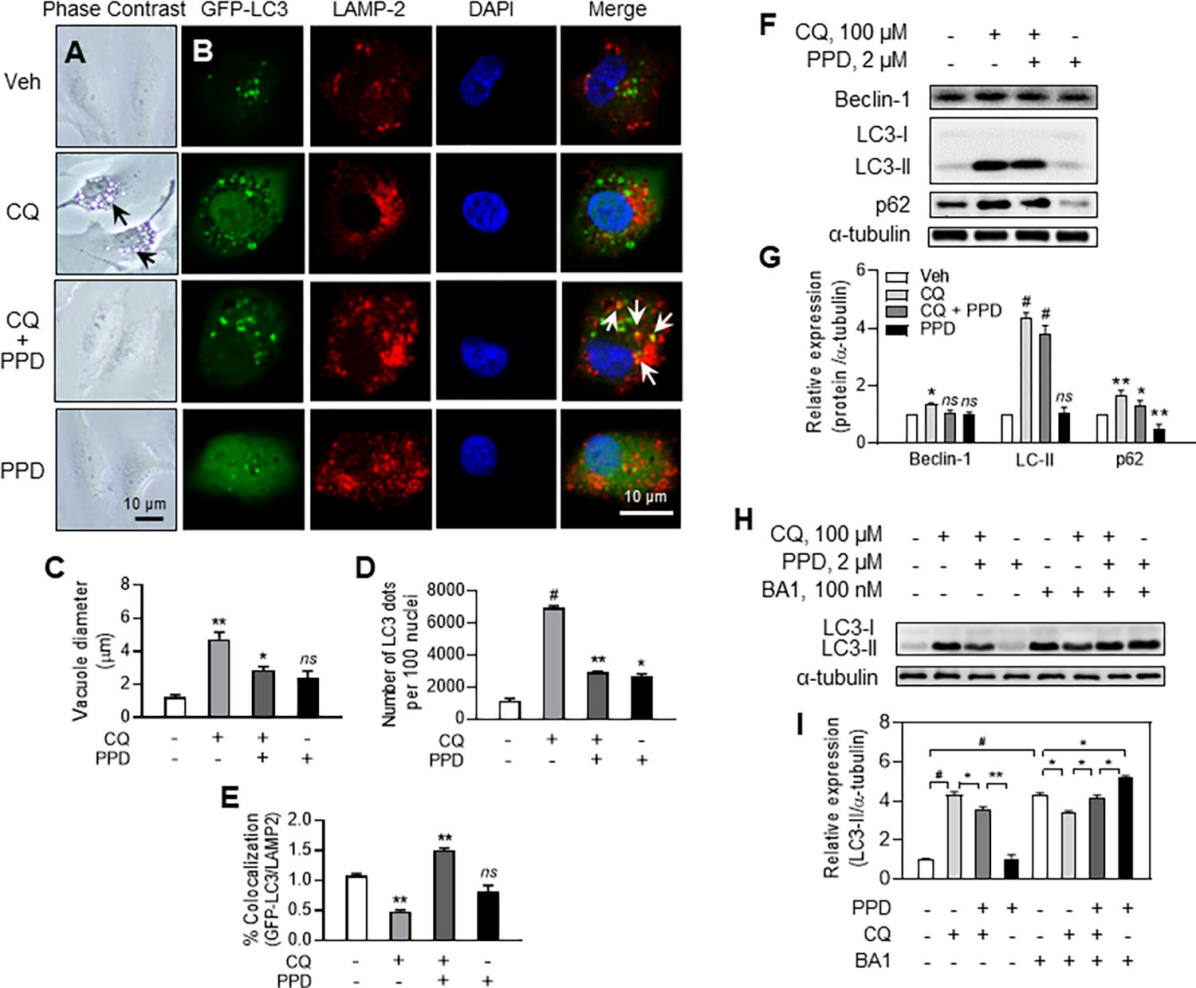
Protopanaxadiol (PPD) enhances liberation of adult retinal pigment epithelial (ARPE)-19 cells from chloroquine-induced autophagy congestion. (A) Phase-contrast photomicrograph of ARPE-19 cells. Large vacuoles in cells treated with CQ for 6 h (black arrows). (B) Confocal images of green fluorescent protein (GFP)-light chain 3 (LC3, green)-transfected cells immunostained with lysosomal membrane-associated protein (LAMP)-2 (red) antibody. Cells treated with vehicle, 100 μM CQ, 2 μM PPD, or 2 μM PPD+100 μM CQ for 6 h. 4’,6-Diamidino-2-phenylindole (blue) counterstained nuclei. Scale bar, 10 μm. Quantification of GFP-LC3-transfected ARPE-19 cells to analyze LC3-positive vacuole (C) size, (D) number (green), and (E) number of LC3-positive (green) vacuoles colocalized (yellow, white arrows) with LAMP-2 (red). (F, H) Western blotting (WB) and (G, I) densitometric analysis of Beclin-1, LC3-II, and p62 in the ARPE-19 cell lysates 6 h after treatment with drugs as in (A). Protein expression levels were compared to levels of α-tubulin and values under blots indicate relative protein expression levels determined by densitometric analysis, unless otherwise specified. Data are means ± standard deviation (SD), n = 3; *ns*; not significant, *P<0.05, **P<0.01 and ^*#*^*P<* 0.001 vs each control.

To determine the potential involvement of PPD in the CQ-induced autophagy defects and lysosomal pH alteration, we used confocal microscopy to examine ARPE-19 cells treated with various drug combinations. Optical microscopy examination revealed that within 6 h of exposure to CQ, APRE-19 cells showed a significant increase in the formation of cytosolic vacuoles, which were larger than those formed in cells treated with the vehicle or PPD alone (Fig 3A, black arrows). However, co-treatment APRE-19 cells with CQ and PPD significantly reduced the vacuole size and number per cell (Fig 3C and 3D).

To identify the large CQ-induced intracellular vacuoles, ARPE-19 cells were induced to overexpress GFP-LC plasmid DNA, which is a protein marker of the autophagosome membrane, and stained with antibodies against LAMP-2, a marker protein localized in the lysosomal membrane (Fig 3B). Treatment with CQ alone induced the formation of both a higher number and larger GFP-LC3 puncta (Fig 3B, CQ) than vehicle treatment did. In addition, the co-localization of LC3 (GFP-LC3, green) and LAMP-2 (red) in ARPE-19 cells treated with CQ alone was not significant (red), suggesting a fusion defect between autophagosomes and lysosomes (Fig 3E). However, co-treatment with PPD and CQ significantly increased the co-localization of these two proteins, with a noticeable decrease in vacuole size (Fig 3A–3C and 3E, CQ+PPD).

### Promotion of autophagy flux by PPD in CQ-treated ARPE-19 cells

To confirm the association of PPD with CQ-induced autophagy defect, we investigated whether autophagy-related cellular signals were altered. WB of Beclin-1, LC3, and p62 from the lysates of ARPE-19 cells exposed to different drug combinations for 6 h showed that CQ increased the expression of LC3, Beclin-1, and p62; however, co-treatment with PPD reduced the expression of these proteins to normal levels (Fig 3F and 3G). PPD alone did not significantly change the expression of Beclin-1 and LC3 levels, but slightly decreased p62 expression (Fig 3F and 3G).

In addition, to determine autophagosome marker protein expression induced by CQ alone or with PPD, an autophagy flux assay was performed using bafilomycin A1 (BA1), a vacuolar H^+^-ATPase (V-ATPase) inhibitor. To measure autophagic flux, it is important to determine the extent of LC3-II degradation in the lysosomes during a certain period. The difference in the amount of LC3-II between groups with and without BA1 represents the level of autophagic flux. Our results with BA1 showed that CQ treatment slightly decreased LC-II expression when compared to basal condition, indicating that CQ suppressed autophagy flux in ARPE-19 cells (Fig 3H and 3I). However, CQ+PPD treatment with BA1 almost completely restored the LC3-II expression to those of BA1 alone-treated cells, and PPD treatment with BA1 highly increased LC3-II expression, suggesting an increased autophagic flux in these cells (Fig 3H and 3I).

### Inhibitory effect of PPD on CQ-induced apoptosis and ROS formation in ARPE-19 cells

**Fig 4:**
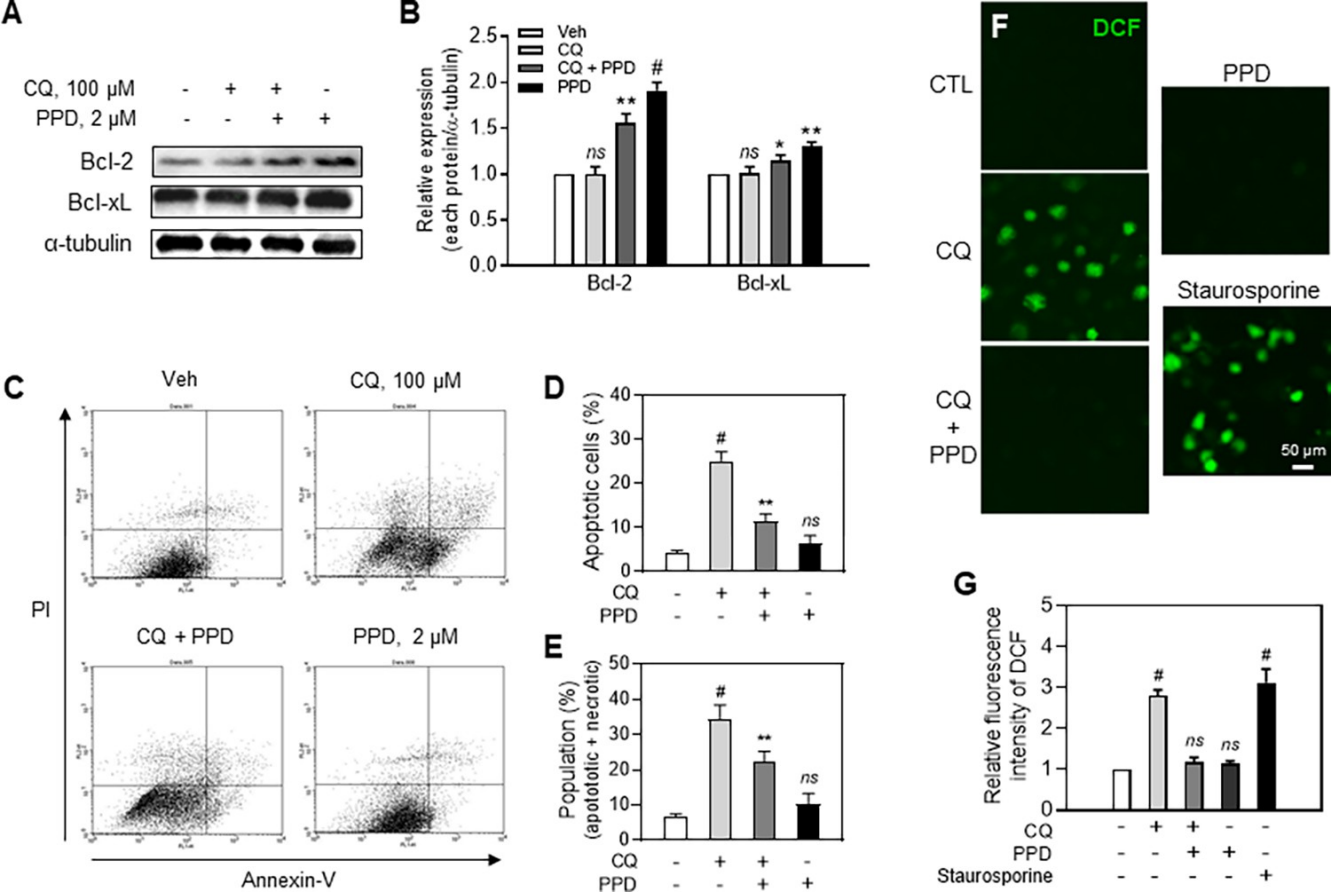
Protopanaxadiol (PPD) protected adult retinal pigment epithelial (APRE)-19 cells from chloroquine-mediated reactive oxygen species (ROS) formation and apoptosis. (A) Western blots and (B) quantitative analysis of Bcl-2 and Bcl-xL in ARPE-19 lysates treated with vehicle, 100 μM CQ, 2 μM PPD+100 μM CQ, and 2 μM PPD for 6 h. (C) Apoptosis assay using flow cytometry and (D, E) quantitative analysis of apoptosis of ARPE-19 cells treated with different drug combinations for 24 h. Bottom left and right show normal and early apoptotic cells, respectively; top right and left show late apoptotic and necrotic cells, respectively. (F) Fluorescence images and (G) relative intensity of DCF-positive fluorescence in ARPE-19 cells. Cells treated with different drug combinations for 24 h were stained with H_2_DCFDA for 1 h. Staurosporine (0.1 μM) was positive control for ROS measurement. Means ± standard deviation (SD), *ns*, *P<0.05, **P<0.01, and ^*#*^*P<* 0.001 vs each control.

To characterize the mechanism underlying the action of PPD on apoptosis, we performed WB for Bcl-2 and Bcl-xL, members of the Bcl-2 family that function as anti-apoptotic factors. CQ did not alter but PPD significantly increased the expression of both proteins, whereas co-treatment with both slightly increased Bcl-2 and Bcl-xL expression levels compared with those of the vehicle-treated cells (Fig 4A and 4B).

Moreover, the FACS analysis (Fig 4C–4E) showed that 24 h treatment with CQ significantly increased early apoptosis (Fig 4C, CQ, lower right column) and necrotic cell death (Fig 4C, CQ, upper left column) by approximately 25% and 9%, respectively. PPD almost completely blocked CQ-induced apoptosis, with an 11% proportion of apoptotic cells (Fig 4D). However, PPD did not restore CQ-induced necrotic cell death, which was still at 9% (Fig 4C). Further examination of the role of ROS in cell death revealed that CQ noticeably increased ROS formation (green signals) in ARPE-19 cells, but PPD completely blocked this effect, suggesting a key role of PPD in ROS-mediated cellular damage (Fig 4F and 4G).

### Regulatory effect of PPD on crosstalk between apoptosis and autophagy via interference with Bcl-2 homology region 3 (BH3)-domain-associated interaction between Beclin-1 and Bcl-2

**Fig 5:**
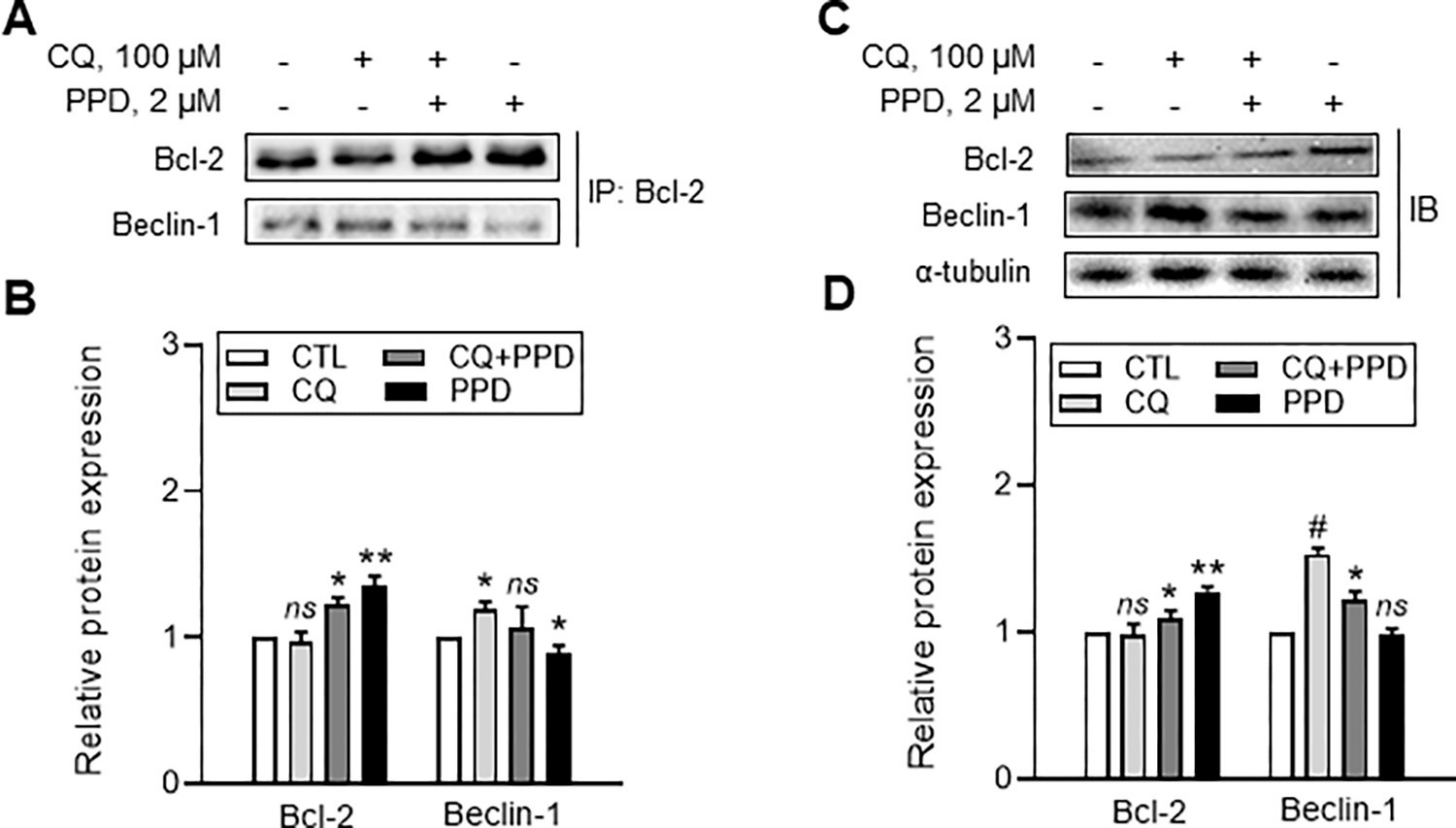
Protopanaxadiol (PPD)-induced alteration on chloroquine-mediated interaction of Beclin-1 BH3 domain with Bcl-2 and cell proliferation associated signaling in adult retinal pigment epithelial (ARPE)-19 cells. ARPE-19 cell lysates (A, B) immunoprecipitated (IP) or (C, D) immunoblotted (IB) with antibodies. Cells were treated with vehicle, 100 μM CQ, 2 μM PPD+100 μM CQ, and 2 μM PPD for 6 h, followed by IP and IB. Data are means ± standard deviation (SD), n = 3; *ns*, *P<0.05, **P<0.01, and ^*#*^*P*<0.001 vs each control.

We examined whether PPD-induced autophagy activation is involved in the interaction between Beclin-1 and Bcl-2. Immunoprecipitation experiments with Bcl-2 antibody showed a strong interaction with Beclin-1 in APRE-19 cells exposed to 100 μM CQ (Fig 5A, CQ). However, this interaction was attenuated by treatment with 2 μM PPD (Fig 5A, CQ+PPD). Moreover, PPD alone induced significantly higher Bcl-2 but lower Beclin 1 levels than those in the vehicle group (Fig 5A and 5B). The analysis of the total protein expression in cell lysates also showed a similar result to that of the immunoprecipitation experiment (Fig 5C and 5D).

### AMPK-mTOR signaling mediated cytoprotective effect of PPD on CQ-treated ARPE-19 cells

**Fig 6:**
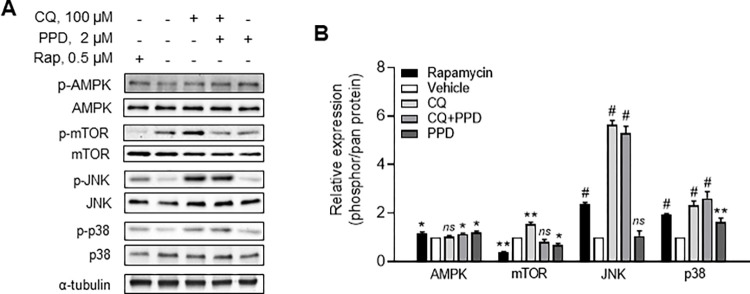
AMP-activated protein kinase (AMPK) and mechanistic target of rapamycin (mTOR) signals are associated with cytoprotective effect of protopanaxadiol (PPD) in chloroquine (CQ)-treated adult retinal pigment epithelial (ARPE)-19 cells. (A) Western blotting (WB) and (B) quantitative analysis of phosphorylated (p)-AMPK, AMPK, p-mTOR, mTOR, p-c-Jun N-terminal kinase (JNK), JNK, p-p38 mitogen-activated protein kinase (MAPK, p-p38), and p38 MAPK (p38) expression in ARPE-19 cells 6 h after treatment with vehicle, 100 μM CQ, 2 μM PPD+100 μM CQ, 2 μM PPD, and 0.5 μM rapamycin (Rap). Data are means ± standard deviation (SD), n = 3; *ns*, *P<0.05, **P<0.01, and ^*#*^*P*<0.001 vs each control.

We further investigated the identified dual effects of PPD on both autophagy and apoptosis by comprehensively examining the potential mediating molecular signals. To achieve this, we focused on upstream and downstream autophagic and cell proliferation signals. The WB revealed that CQ increased the phosphorylation of mTOR, JNK, and p38 but not that of AMPK (Fig 6). However, co-treatment with PPD did not alter the CQ-mediated induction of JNK and p38, but significantly decreased mTOR activity and increased AMPK activity (Fig 6).

**Fig 7:**
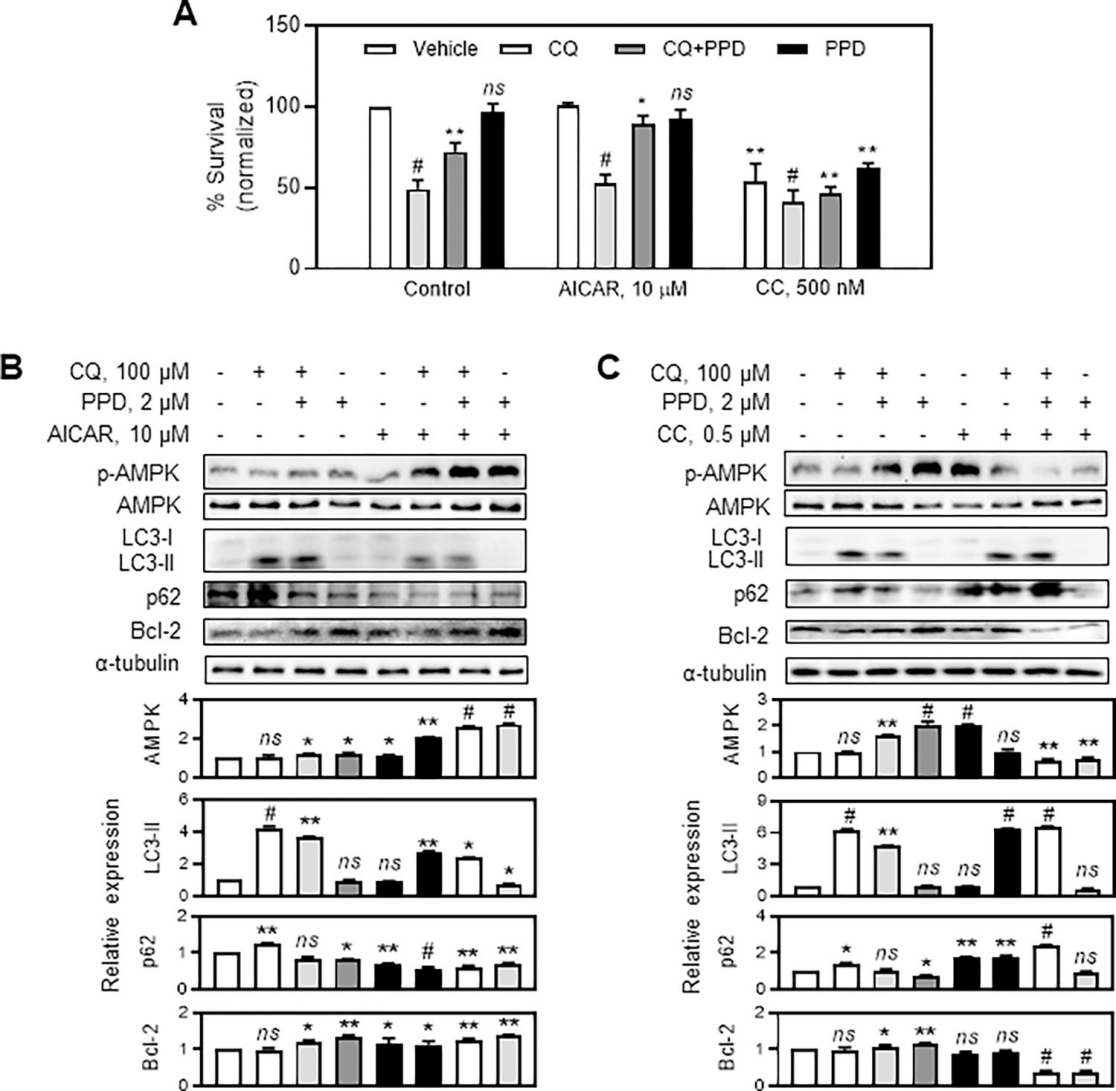
Modulation of AMP-activated protein kinase (AMPK)-induced alteration of protopanaxadiol (PPD) effects on chloroquine (CQ)-treated adult retinal pigment epithelial (ARPE)-19 cells. (A) Relative survival rate of ARPE-19 cells treated with vehicle, 100 μM CQ, CQ+PPD, and 2 μM PPD with vehicle, 10 μM AICAR, or 500 nM Compound C for 24 h. Cytotoxicity was assessed using 3-(4,5-dimethyl-thiazol-2-yl)-2,5-diphenyltetrazolium bromide (MTT) assay. (B, C) Western blotting (WB) and quantitative analysis of phosphorylated (p)-AMPK, AMPK, light chain 3 (LC3) I/II, p62, and B-cell lymphoma 2 (Bcl-2). Cells were treated with various drug combinations as indicate in (A) for 6 h. Bars denote relative changes in each protein. Data are means ± standard deviation (SD), n = 3; *ns*, *P<0.05, **P<0.01, and ^*#*^*P*<0.001 vs control vehicle.

PPD increased AMPK activity and therefore, we determine whether AMPK activity plays a key role in mediating its effects in CQ-treated ARPE-19 cells. The cells were co-treated with the AMPK activator AICAR (10 μM) or AMPK inhibitor Compound C (0.5 μM) with vehicle, 100 μM CQ, 2 μM PPD+100 μM CQ, and 2 μM PPD, and cytotoxicity and cellular signaling changes were determined. As shown in Fig 7A, AICAR strengthened the protective effects of PPD on CQ-treated ARPE-19 cells, but Compound C, significantly weakened the PPD-mediated cytoprotection. Moreover, the WB demonstrated that AMPK activation by AICAR significantly enhanced the PPD-mediated AMPK, LC3-II, p62, and Bcl-2 alterations, but suppression of AMPK by Compound C abolished these effects (Fig 7B and 7C).

**Fig 8:**
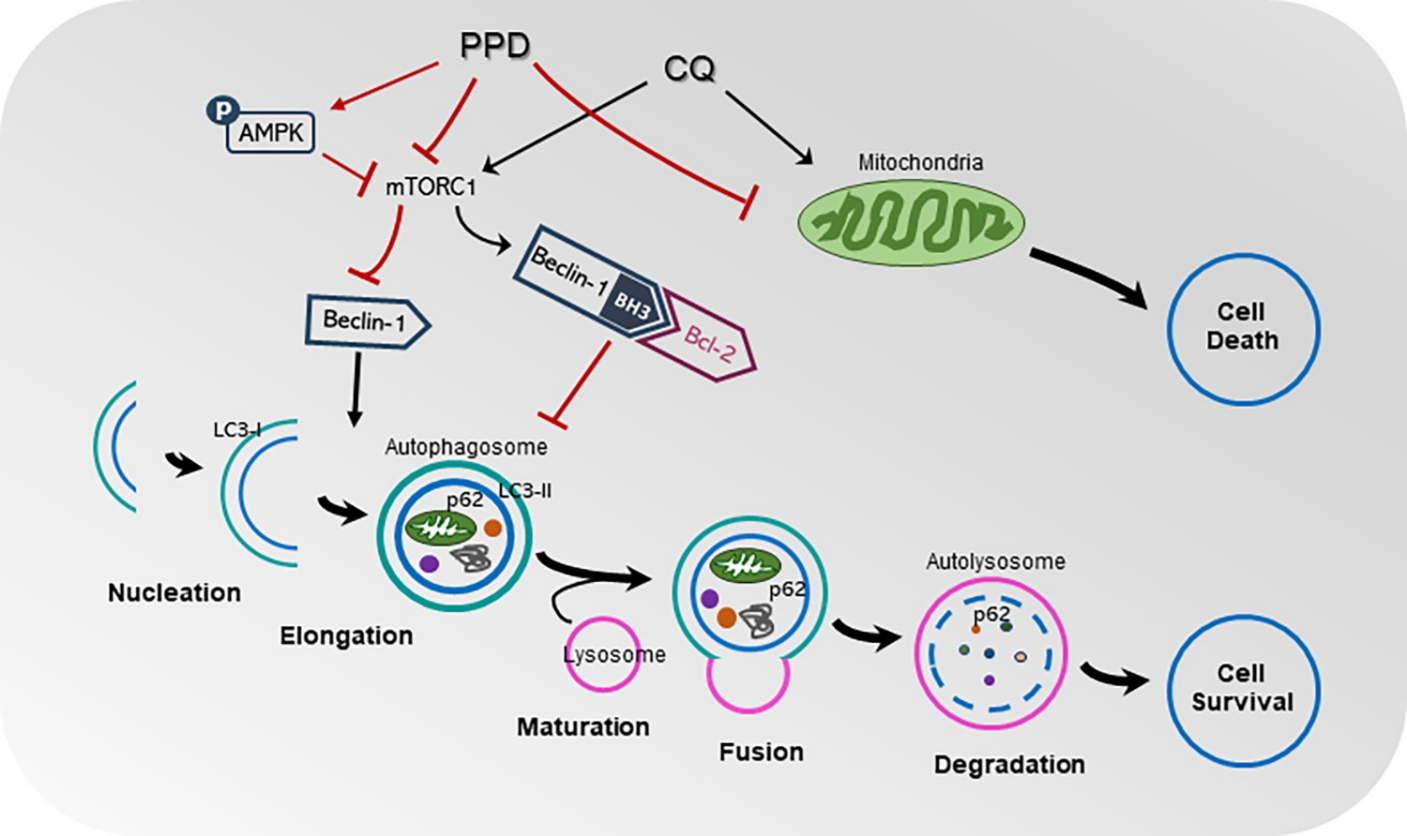
Schematic representation of effect of protopanaxadiol (PPD) on crosstalk between autophagy and apoptosis in chloroquine-treated adult retinal pigment epithelial (ARPE)-19 cells. PPD activated AMP-activated protein kinase (AMPK) activity but inhibited CQ-induced increase of mechanistic target of rapamycin (mTORC) activity and anti-apoptotic protein B-cell lymphoma 2 (Bcl-2) expression. This action altered CQ-mediated interactions between Beclin-1 and Bcl-2 via Bcl-2 homology region 3 (BH3) domain, to activate Beclin-1. Beclin-1, LC3, and p62 are associated with autophagosome formation and autophagy flux. PPD may act critically on gateway of autophagy and apoptosis intersection in mediating CQ-treated ARPE-19 cell toxicity, restoring normal cellular homeostasis.

The overall sequence of the reactions still needs to be clarified, but we confirmed that the regulatory action of PPD on both autophagy and apoptosis was mediated through the inhibition of mTOR and activation of AMPK. In particular, the control of the interaction between Beclin-1 and Bcl-2 was a key factor in this effect. PPD interfered with the interaction between Beclin-1 and Bcl-2 via its BH3 domain, and thereby promoted the release of Beclin-1 from Bcl-2. Because Beclin-1 is closely associated with autophagosome formation, these reactions may have contributed to increasing autophagy in CQ-treated ARPE-19 cells (Fig 8).

## Discussion

In this study, we demonstrated that PPD protected ARPE-19 cells against CQ-induced cytotoxicity, which was likely mediated mainly by autophagy and apoptosis. The initial change observed in human RPE-derived ARPE-19 cells after treatment with CQ was the accumulation of enlarged autophagosomes and lysosomes and an increase in intralysosomal pH. In practice, the RPE regularly engulfs the damaged outer segment of photoreceptors into the lysosomes, which regenerates the photoreceptors. Therefore, damage to the RPE is central to disease progression.

LC3-II in the inner membrane is rapidly degraded when autophagosomes fuse with lysosomes, whereas that in the outer membrane remains intact until it is recycled [20]. Both autophagy activation and inhibition greatly increase LC3-II levels; therefore, the LC3-II level at any particular time is not necessarily an indication of autophagic activity [21]. Consistently, the autophagosomes that accumulated in CQ-treated ARPE-19 cells did not fuse with lysosomes and caused local derangement.

Hence, the induction of Beclin-1 and LC3-II is likely a secondary effect of fusion failure, rather than an indicator of autophagy activation, which is toxic to the RPE. In this study, PPD did not significantly change the basal levels of LC3-II, but promoted expression of LC-II in the presence of BA1, indicating an increase in autophagy flux. PPD also appeared to control autophagy extensively because it significantly altered CQ-induced Beclin-1 expression, an autophagy marker for the early stages of autophagosome formation, in addition to p62 and LC3-II, which are markers for the late stages associated with autolysosome formation.

Numerous studies have reported the effects of various ginseng extracts on autophagy, but the underlying mechanisms mediating these functional effects remain controversial. For example, ginsenosides Rb1 and F2 increase autophagy in bronchoalveolar [22] and breast cancer cells [23], whereas ginsenosides Rg3 and Compound C inhibit autophagy in hepatocellular carcinoma [24] and neuroblastoma cells [19], respectively. Thus, depending on the cell type or disease characteristics, the same autophagy activation could exhibit different effects such as cytotoxicity or cytoprotection.

In addition to autophagy, apoptosis also plays a pivotal role in cell fate under both normal cellular metabolic and stress conditions [25]. In the present study, contrary to a change in autophagy activity, CQ induced the apoptosis of ARPE-19 cells through the inhibition of Bcl-2 and Bcl-xL, but this change was attenuated by PPD. ROS also appeared to be involved in the CQ-induced RPE toxicity. CQ entering the lysosome increases the lysosomal pH, leading to the simultaneous release of Fe^2+^ and a reduction in lysosomal hydrolase activity [26]. These effects cause Fenton’s reaction and oxidative stress in the cells via ROS formation [26, 27]. We did not examine in detail whether the CQ-induced ROS increase in ROS directly activated apoptosis. However, CQ-induced apoptosis of ARPE-19 cells may concomitantly occur following ROS increase, and PPD abolished all the effects induced by CQ.

Cell survival and death signaling pathways are closely connected, and there is no one-way path that determines cell fate. Autophagy and apoptosis, which are two distinct processes that play opposing biological roles in the response to diverse stresses, are triggered by common upstream signals. Beclin-1 was recently classified as a BH3-only protein in studies using a yeast two-hybrid screen and was first identified to strongly bind to Bcl-2 and Bcl-xL, and this interaction reduces Beclin-1-mediated autophagy induction [11, 28, 29], but Beclin-1 bound Bcl-2 maintains full anti-apoptotic function [30]. Similarly, Bcl-2 interferes with the formation of autophagy-promoting Vps34/Beclin-1 complex [31]. This observation suggests that the transient interaction between Bcl-2 and Beclin-1 enables flexible and dynamic regulation of autophagy induction.

In this study, the CQ enhanced binding affinity of Bcl-2 and Beclin-1 in ARPE-19 cells was inhibited by PPD. Our findings also prove that Bcl-2 likely inhibits pro-autophagic action of Beclin-1 by arrest Beclin-1’s activity. During this process, PPD specifically interrupted the CQ-induced association of Bcl-2 and Beclin-1 and, consequently, protected the cells against detrimental effects.

Phosphoinositide 3-kinase/mTOR and AMPK, the main signaling molecules associated with autophagy, are highly sensitive to internal nutrient conditions by monitoring the cellular AMP:ATP ratio [32]. In addition, oxidative stress is related to these signaling molecules, suggesting a possible crosslinking with ROS-inducible apoptosis. Suppression of mTOR activity via AMPK is closely associated with autophagy induction.

In particular, AMPK regulates the function of the Beclin-1 complex that includes VPS34, which is essential for autophagosome formation [33]. Interestingly, in the present study, CQ increased mTOR phosphorylation but did not alter AMPK activity, implying that CQ had no direct effect on AMPK. However, co-treatment with PPD noticeably activated AMPK phosphorylation, followed by the recovery of mTOR activity, which was increased by CQ to almost normal levels. Similarly, the AMPK activator, AICAR, enhanced the effect of PPD.

Several studies have reported that AMPK activity is also linked to the stress-activated MAPK signals of p38 MAPK and JNK, which are highly phosphorylated under stress conditions [34–36]. Moreover, several ginsenoside derivatives have been reported to control p38 MAPK or JNK-induced apoptosis, as well as autophagy [37–39]. Both p38 MAPK and JNK promote Bcl-2 phosphrylation, leading to the interruption and dissociation of the Belcin-1-Bcl-2 complex, and the liberation of Belcin-1 initiates autophagy [40]. However, continous activation of Bcl-2 induces the steady accumulation of multi-site phosphorylated Bcl-2, which interferes with the protective effects of autopahgy and leads to cytotoxicity [41]. Similar examinations of CQ in ARPE-19 cells shows that it significantly activated p38 MAPK and JNK, but PPD did not further change this activation. This observation suggests that p38 MAPK and JNK are not involved in the PPD-mediated switch between autophagy and apoptosis.

## Conclusion

In conclusion, several aspects of the underlying mechanisms of action of PPD still need to be comprehensively elucidated, such as the identity of the main signaling molecule that directs the PPD-mediated rescue of CQ-induced ARPE-19 toxicity. In addition, other outstanding activities of interest include the cellular biomolecules associated with the PPD-autophagy-apoptosis axis, the mechanism by which AMPK communicates with Beclin-1 or Bcl-2, and whether any correlation exist between different serine/threonine protein kinases when cells react to CQ. Nevertheless, our current findings suggest that PPD may be a therapeutically attractive and useful candidate drug for CQ-induced retinopathy, warranting further investigation for potential clinical development.

## Supporting information

S1 Raw images Original blot collection for western blots included in the entire figures. (TIF)
