# Supplementary figures and images for: Ginsenoside protopanaxadiol protects adult retinal pigment epithelial-19 cells from chloroquine by modulating autophagy and apoptosis
Source: PLoS One. 2022 Dec 1;17(12):e0274763. doi: 10.1371/journal.pone.0274763 (PMC9714852; doi:10.1371/journal.pone.0274763)

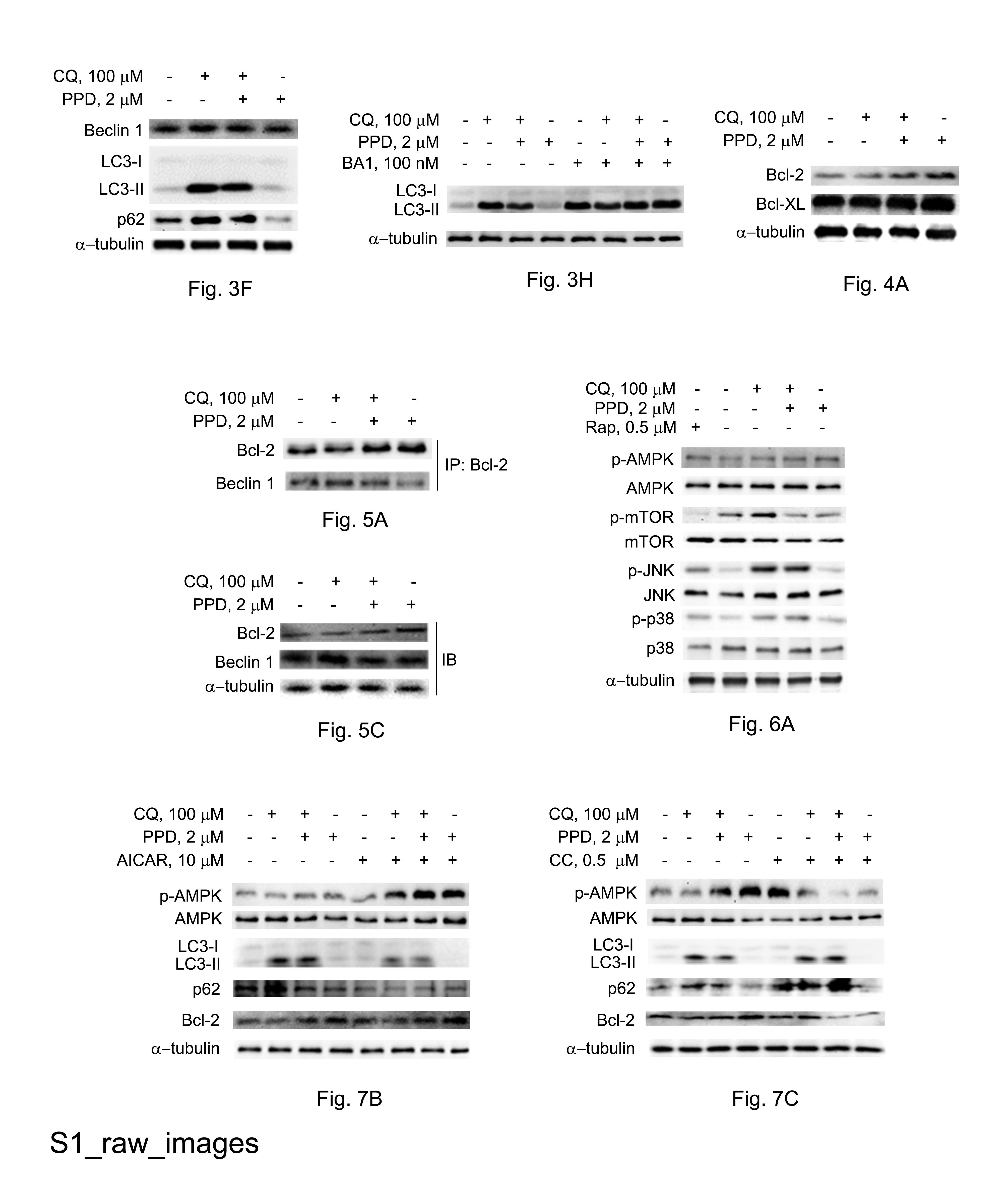

Supplement: S1 Raw images — (TIF) [file pone.0274763.s001.tif]
